# Supplementary material for: Glucocerebrosidase activity and lipid levels are related to protein pathologies in Parkinson’s disease
Source: NPJ Parkinsons Dis. 2023 May 11;9:74. doi: 10.1038/s41531-023-00517-w (PMC10175254; doi:10.1038/s41531-023-00517-w)
Supplement: Supplementary file 1 — Supplementary Material [file 41531_2023_517_MOESM1_ESM.pdf]

Supplementary materials for

Glucocerebrosidase activity and lipid levels are related to protein pathologies in Parkinson's disease

Cheryl E. G. Leyns[2], Alice Prigent[1], Brenna Beezhold[1], Lihang Yao[3], Nathan G. Hatcher[3], Peining Tao[2], John Kang[2], EunRan Suh[4], Vivianna M. Van Deerlin[4], John Q. Trojanowski[4], Virginia M.Y. Lee[4], Matthew E. Kennedy[2], Matthew J. Fell[2], Michael X. Henderson[1]*

Supplementary Table 1

Group	Subgroup	Mutation	Race	Ethnicity	Sex	Age	Disease Duration (y)
Control	Unremarkable	None	White	Not Hispanic or Latino	Male	61	NA
Control	Unremarkable	None	White	Not Hispanic or Latino	Male	70	NA
Control	Unremarkable	None	Black	Not Hispanic or Latino	Male	55	NA
Control	Unremarkable	None	Black	Not Hispanic or Latino	Female	59	NA
Control	Unremarkable	None	White	Not Hispanic or Latino	Female	65	NA
Control	Unremarkable	None	White	Not Hispanic or Latino	Male	85	NA
Control	Unremarkable	None	White	Not Hispanic or Latino	Female	63	NA
Control	Unremarkable	None	White	Not Hispanic or Latino	Male	62	NA
Control	Unremarkable	None	White	Not Hispanic or Latino	Male	57	NA
Control	Unremarkable	None	White		Male	59	NA
Control	Unremarkable	None	White		Female	56	NA
Control	Unremarkable	None	Black		Male	62	NA
Control	Unremarkable	None	White		Male	52	NA
Control	Unremarkable	None	White		Male	71	NA
Control	Unremarkable	None	White		Male	68	NA
Control	Unremarkable	None	Black		Male	69	NA
Control	Unremarkable	None	Black		Male	66	NA
Control	Unremarkable	None	White		Female	60	NA
Control	Unremarkable	None	White		Male	54	NA
Idiopathic	iPD	None	White	Not Hispanic or Latino	Female	80	11
Idiopathic	iPD	None	White	Not Hispanic or Latino	Male	63	6
Idiopathic	iPD	None	White	Not Hispanic or Latino	Female	76	NA
Idiopathic	iPD	None	White	Not Hispanic or Latino	Male	86	26
Idiopathic	iPD	None	White	Not Hispanic or Latino	Female	70	18
Idiopathic	iPD	None	White	Not Hispanic or Latino	Male	58	8
Idiopathic	iPD	None	White	Not Hispanic or Latino	Female	77	11
Idiopathic	iPD	None	White		Male	62	9
Idiopathic	iPD	None	White	Not Hispanic or Latino	Male	81	12
Idiopathic	iPD	None	White	Not Hispanic or Latino	Male	84	19
Idiopathic	iPD	None	White		Female	79	20
Idiopathic	iPD	None	White	Not Hispanic or Latino	Female	72	22
Idiopathic	iPDD	None	White	Not Hispanic or Latino	Male	78	25
Idiopathic	iPDD	None	White		Male	79	9
Idiopathic	iPDD	None	White	Not Hispanic or Latino	Male	87	15
Idiopathic	iPDD	None	White		Male	64	17
Idiopathic	iPDD	None	White	Not Hispanic or Latino	Male	73	14
Idiopathic	iPDD	None	White	Not Hispanic or Latino	Male	87	28
Idiopathic	iPDD	None	White	Not Hispanic or Latino	Male	63	23
Idiopathic	iPDD	None	White	Not Hispanic or Latino	Male	71	7

Idiopathic	iPDD	None	White	Not Hispanic or Latino	Male	59	20
Idiopathic	iPDD	None	White	Not Hispanic or Latino	Male	83	12
Idiopathic	iPDD	None	White	Not Hispanic or Latino	Female	64	21
Idiopathic	iPDD	None	White	Not Hispanic or Latino	Female	91	13
Idiopathic	iPDD	None	White	Not Hispanic or Latino	Male	68	8
Idiopathic	iPDD	None	White		Male	91	8
Idiopathic	iDLB	None	White	Not Hispanic or Latino	Male	66	4
Idiopathic	iDLB	None	White	Not Hispanic or Latino	Male	71	9
Idiopathic	iDLB	None	White	Not Hispanic or Latino	Male	77	8
Idiopathic	iDLB	None	White	Not Hispanic or Latino	Male	80	9
Idiopathic	iDLB	None	White	Not Hispanic or Latino	Male	71	9
Idiopathic	iDLB	None	White	Not Hispanic or Latino	Male	68	8
Idiopathic	iDLB	None			Male	62	NA
Idiopathic	iDLB	None	White	Not Hispanic or Latino	Male	70	6
Idiopathic	iDLB	None	White	Not Hispanic or Latino	Female	81	13
Idiopathic	iDLB	None	White	Not Hispanic or Latino	Female	69	5
Idiopathic	iDLB	None	White	Not Hispanic or Latino	Male	67	5
GBA1	*GBA1*-PD	GBA (A456P)	White	Not Hispanic or Latino	Male	76	8
GBA1	*GBA1*-PD	GBA (N370S)	White	Not Hispanic or Latino	Male	70	17
GBA1	*GBA1*-PD	GBA (L444P)	White	Not Hispanic or Latino	Female	72	26
GBA1	*GBA1*-PD	GBA (L444P)	White	Not Hispanic or Latino	Male	66	10
GBA1	*GBA1*-PDD	GBA (S196P)	White		Female	66	16
GBA1	*GBA1*-PDD	GBA (L444P)	White	Not Hispanic or Latino	Male	73	10
GBA1	*GBA1*-PDD	GBA (N370S)	White		Male	83	14
GBA1	*GBA1*-PDD	GBA (N370S)	White	Not Hispanic or Latino	Male	71	10
GBA1	*GBA1*-PDD	GBA (N370S)	White	Not Hispanic or Latino	Male	86	10
GBA1	*GBA1*-PDD	GBA (N370S)	White	Not Hispanic or Latino	Male	67	9
GBA1	*GBA1*-PDD	GBA Rec1	Black	Not Hispanic or Latino	Male	61	9
GBA1	*GBA1*-PDD	GBA (N370S, R463C)	White	Not Hispanic or Latino	Male	67	13
GBA1	*GBA1*-PDD	GBA (N370S)	White	Not Hispanic or Latino	Female	92	33
GBA1	*GBA1*-PDD	GBA (N370S)	White	Not Hispanic or Latino	Male	79	14
GBA1	*GBA1*-PDD	GBA (N370S)	White	Not Hispanic or Latino	Male	78	7
GBA1	*GBA1*-PDD	GBA (R359X)	White	Not Hispanic or Latino	Male	58	21
GBA1	*GBA1*-PDD	GBA (N370S)	White	Not Hispanic or Latino	Male	68	17
GBA1	*GBA1*-PDD	GBA Rec1	White		Male	72	6
GBA1	*GBA1*-DLB/AD	GBA (N370S)	White	Not Hispanic or Latino	Male	68	7
GBA1	*GBA1*-DLB/AD	GBA (N370S)	White	Not Hispanic or Latino	Male	59	8
GBA1	*GBA1*-DLB/AD	GBA (N370S)	White	Not Hispanic or Latino	Female	88	10
GBA1	*GBA1*-DLB/AD	GBA (N370S)	White	Not Hispanic or Latino	Male	81	10
GBA1	*GBA1*-DLB/AD	GBA (N370S)	White	Not Hispanic or Latino	Male	90	5
GBA1	*GBA1*-DLB/AD	GBA (A456P)	White	Not Hispanic or Latino	Male	77	8

GBA1	*GBA1*-DLB/AD	GBA (N370S) Homo	White	Not Hispanic or Latino	Male	70	9
GBA1	*GBA1*-DLB/AD	GBA (N370S)	White	Not Hispanic or Latino	Male	71	4
GBA1	*GBA1*-DLB/AD	GBA (N370S)	White	Not Hispanic or Latino	Female	80	NA
GBA1	*GBA1*-DLB/AD	GBA (N370S)	White		Male	60	9
LRRK2	LRRK2-PD	LRRK2 (G2019S)	White		Male	86	19
LRRK2	LRRK2-PD	LRRK2 (G2019S)	White		Male	84	19
LRRK2	LRRK2-PD	LRRK2 (G2019S)	White		Female	77	5
LRRK2	LRRK2-PD	LRRK2 (G2019S) Homo	White	Not Hispanic or Latino	Female	79	31
LRRK2	LRRK2-PDD	LRRK2 (G2019S)	White	Not Hispanic or Latino	Male	81	28
LRRK2	LRRK2-PDD	LRRK2 (L1165P)	White	Not Hispanic or Latino	Male	81	36
LRRK2	LRRK2-PDD	LRRK2 (R793M)	White		Female	92	15

Supplementary Table 1. Case variant and demographic information. For each individual case, the specific variant and demographic information is shown.

Study	Groups	Brain Regions	GCase Activity *GBA1*-PD	GCase Activity iPD	Lipid Levels GlcCer	Lipid Levels GlcSph	Path.
Gegg et al. (2012)	**HC:** 6-10<br>**iPD:** 12-14<br>***GBA1*-PD:** 9-14	Cerebellum	↓	↓	ND	ND	ND
		Frontal	–	–	ND	ND	ND
		Putamen	↓	–	ND	ND	ND
		Amygdala	↓	–	ND	ND	ND
		Substantia nigra	↓	↓	ND	ND	ND
Murphy et al. (2014)	**HC:** 10<br>**iPD:** 19	Cingulate	ND	↓	ND	ND	α-Syn Biochemistry
		Occipital	ND	–	ND	ND	
Gegg et al. (2015)	**HC:** 5-7<br>**iPD:** 7-13<br>***GBA1*-PD:** 5-14	Cerebellum	ND	ND	–	ND	ND
		Putamen	ND	ND	–	ND	ND
Rocha et al. (2015)	**HC:** 29<br>**iPD:** 25	Cerebellum	ND	↓	ND	–	ND
		Frontal	ND	–	ND	–	ND
		Putamen	ND	↓	ND	–	ND
		Hippocampus	ND	↓	ND	↑	ND
		Substantia nigra	ND	↓	ND	↑	ND
			*Different by age		*Different by age		
Boutin et al. (2016)	**HC:** 12<br>**iPD:** 32	Temporal	ND	ND	–	ND	ND
Huebecker et al. (2019)	**HC:** 10-18<br>**iPD:** 7-14<br>***GBA1*-PD:** 3-4	Substantia nigra	–	↓	↑	↑	ND
			*Different by age		*Different by age		
Gunder et al. (2019)	**HC:** 15<br>**iPD:** 20<br>***GBA1*-PD:** 10	Frontal	ND	–	ND	↑	α-Syn Biochemistry
		Putamen	ND	–	ND	–	
		Substantia nigra	ND	–	ND	–	
Kurzawa-Akanbi et al. (2021)	**HC:** 16<br>***GBA1*-HC:** 10<br>**iPD:** 17<br>***GBA1*-PD:** 21	Frontal	ND	ND	–	ND	ND
		Cingulate	ND	ND	–	ND	ND
Blumenreich et al. (2022)	**HC:** 21<br>**iPD:** 21<br>***GBA1*-PD: 21**	Middle Temporal	ND	ND	↑	ND	ND
		Cingulate	ND	ND	–	ND	ND
		Striatum	ND	ND	–	ND	ND
		Occipital	ND	ND	–	ND	ND
Current Study	**HC:** 18<br>**iPD:** 37<br>***GBA1*-PD:** 28<br>***LRRK2*-PD:** 7	Frontal	↓	–	–	↑	**IHC:**<br>α-Syn, Tau, Aβ,TDP-43
		Cingulate	↓	–	–	↑↑	
		Putamen	↓	–	–	↑	
		Cerebellum	↓	–	–	↑	

Supplementary Figure 1. Summary of GCase and lipid studies in human brain Findings from previous studies that have examined GCase activity, GlcCer or GlcSph lipid levels or pathological proteins in idiopathic or GBA-PD are summarized here. The current study is listed last on the table. ND indicates that this measure was "Not Determined." Hyphens indicate no significant change in this measure. Down arrows indicate a decrease. Up arrows indicate an increase. The two up arrows in the current study are to indicate the increase of GlcSph in both idiopathic as well as *GBA1*-PD.

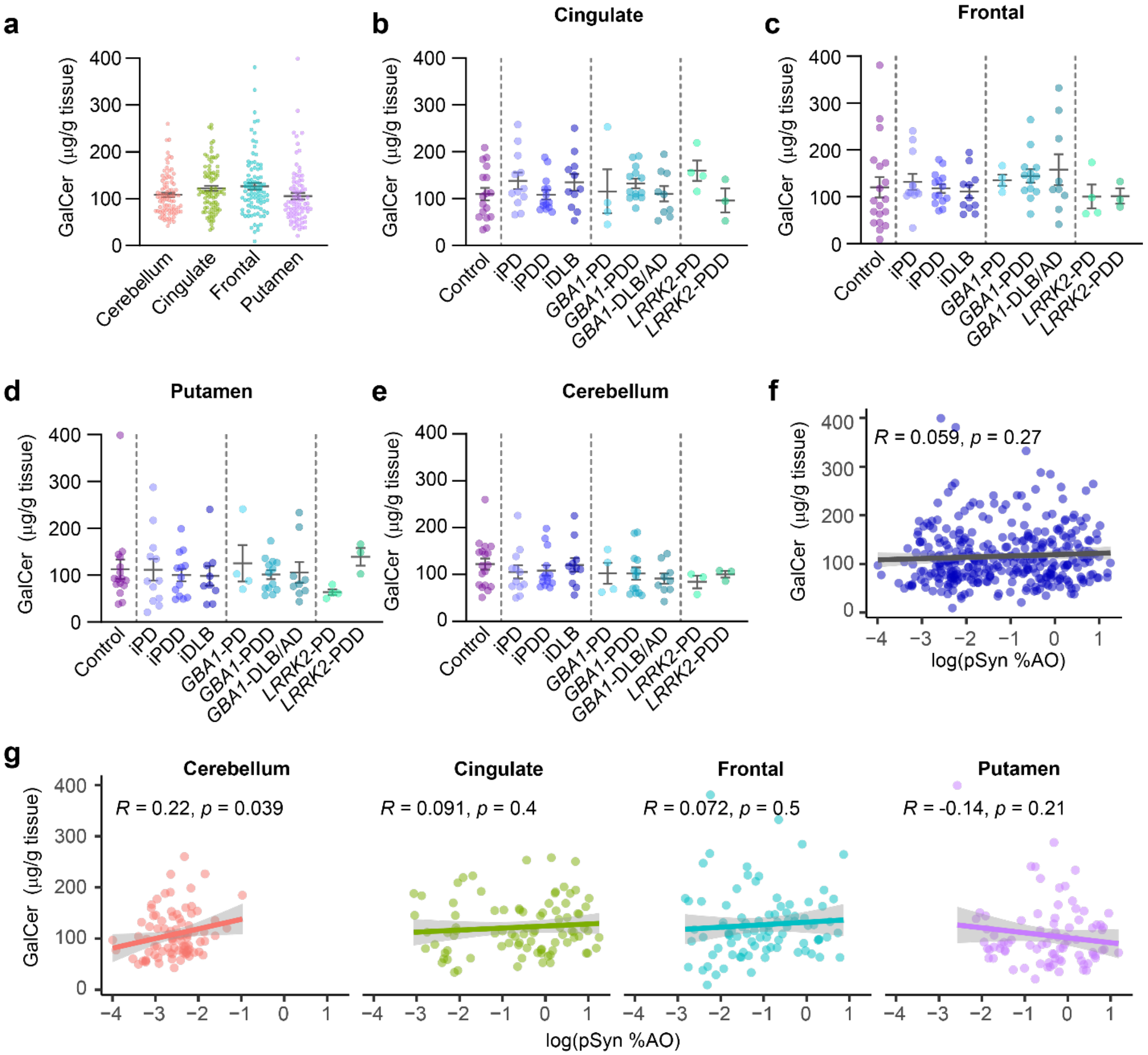

Supplementary Figure 2. GalCer in genetic and idiopathic PD (**a**) Total GalCer measures for all cases, separated by region. GalCer levels are subsequently broken down by neuropathological disease and genetics for each of the four regions: (**b**) cingulate, (**c**) frontal, (**d**) putamen, and (**e**) cerebellum. Bars represent mean +/- S.E.M. with individual values plotted. (**f**) Log normalized pSyn pathology plotted against GalCer levels for all samples. (**g**) Log normalized pSyn pathology plotted against GalCer levels but broken down by brain region. Lines represent linear regression line of best-fit and shaded area is the 95% confidence interval. Panels **a**, **b**, **c**, **d**, **e**: One-way ANOVA; Tukey's multiple comparison test.

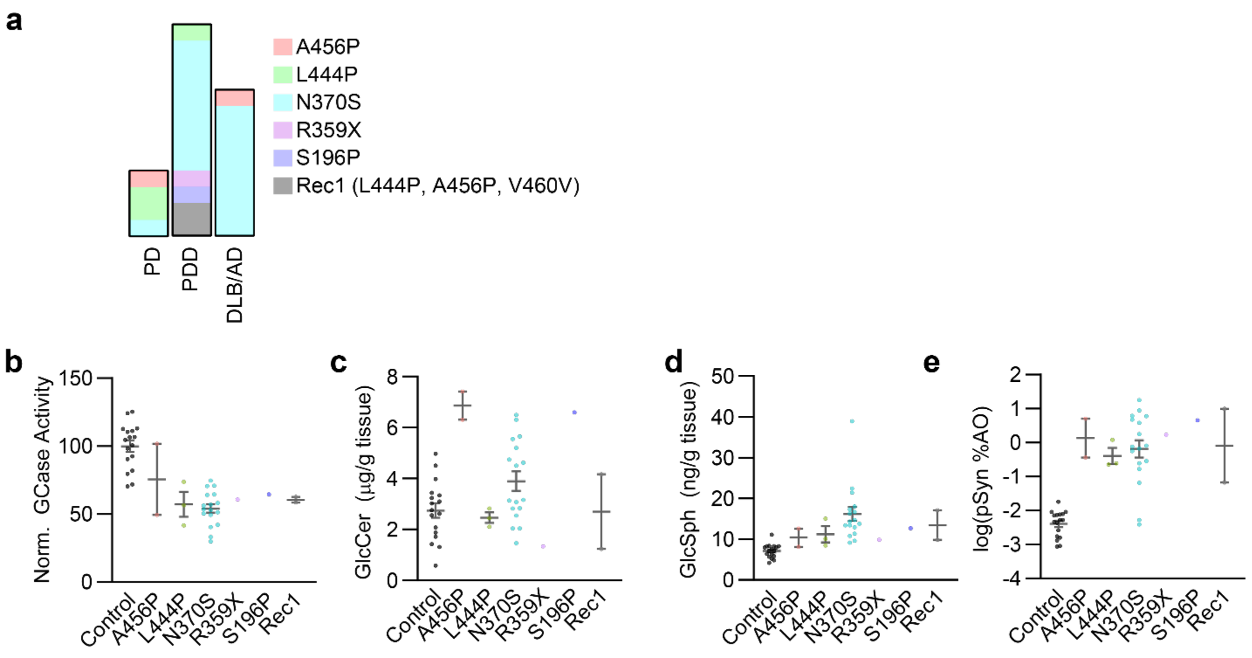

Supplementary Figure 3. *GBA1* mutation analysis (**a**) The proportion of all *GBA1* mutation carriers with the noted variant in each disease category is noted. N370S was the predominant mutation overall, and especially for PDD and DLB/AD groups. The PD group was small, but two out of four cases carried L444P. (**b**) Normalized GCase activity in the cingulate cortex for control and *GBA1* mutation carriers. (**c**) Total GlcCer levels in the cingulate cortex for control and *GBA1* mutation carriers. (**d**) GlcSph levels in the cingulate cortex for control and *GBA1* mutation carriers. (**e**) pSyn levels in the cingulate cortex of control and *GBA1* mutation carriers. The number of carriers for individual mutations was insufficient to make statistical comparisons, but no major differences were observed by genotype. Bars represent mean +/- S.E.M. with individual values plotted.

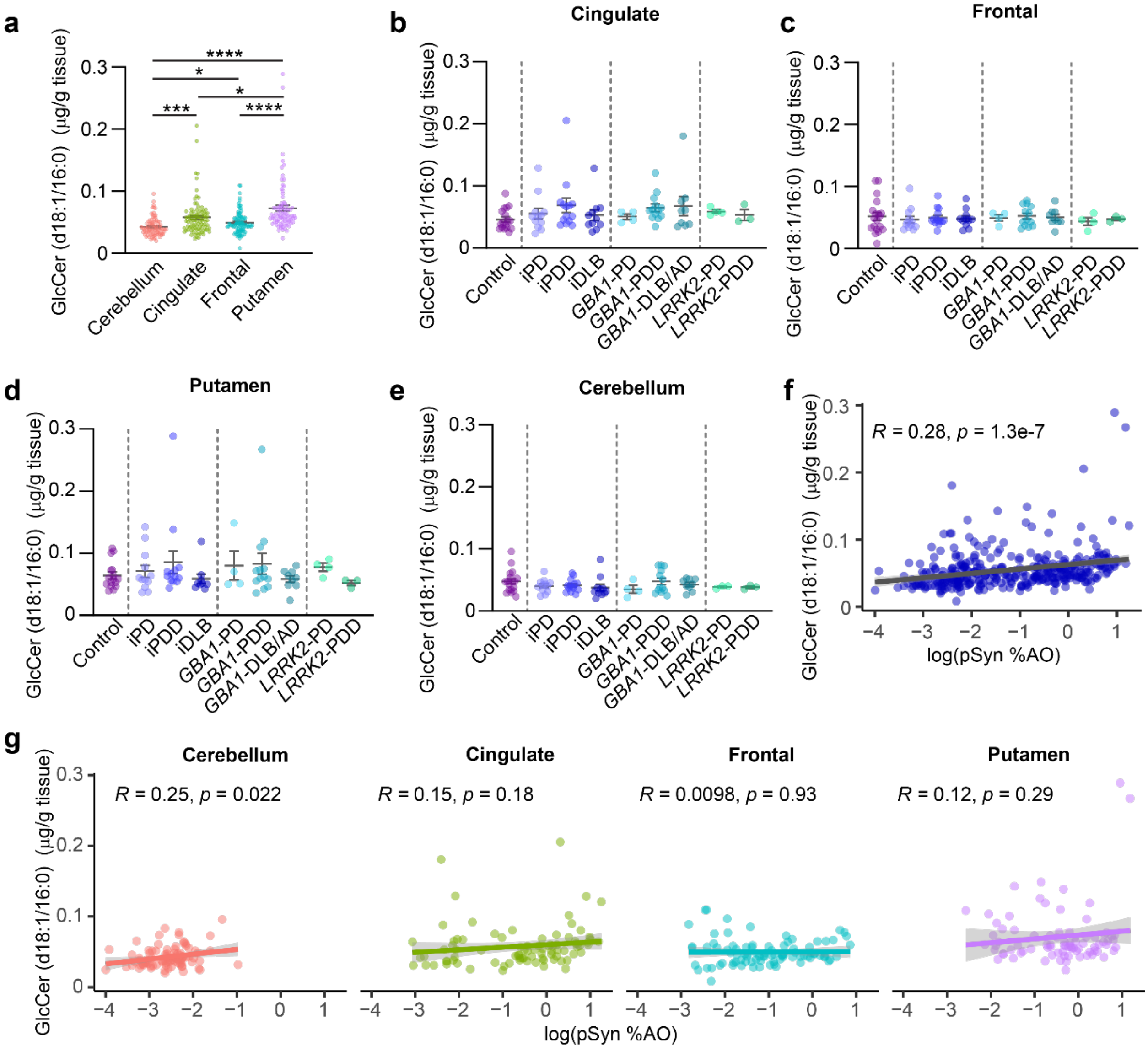

Supplementary Figure 4. GlcCer (d18:1/16:0) isoform in genetic and idiopathic PD (**a**) GlcCer (d18:1/16:0) measures for all cases, separated by brain region. GlcCer species levels are subsequently broken down by neuropathological disease and genetics for each of the four regions: (**b**) cingulate, (**c**) frontal, (**d**) putamen, and (**e**) cerebellum. Bars represent mean +/- S.E.M. with individual values plotted. (**f**) Log normalized pSyn pathology plotted against normalized GlcCer (d18:1/16:0) activity for all samples. (**g**) Log normalized pSyn pathology plotted against GlcCer (d18:1/16:0) levels but broken down by brain region. Lines represent linear regression line of best-fit and shaded area is the 95% confidence interval. Panel **a**: Welch's ANOVA test; Dunnett's T3 multiple comparisons test. **b**, **c**, **d**, **e**: One-way ANOVA; Tukey's multiple comparison test. *p<0.05, **p<0.01, ***p<0.001, ****p<0.0001.

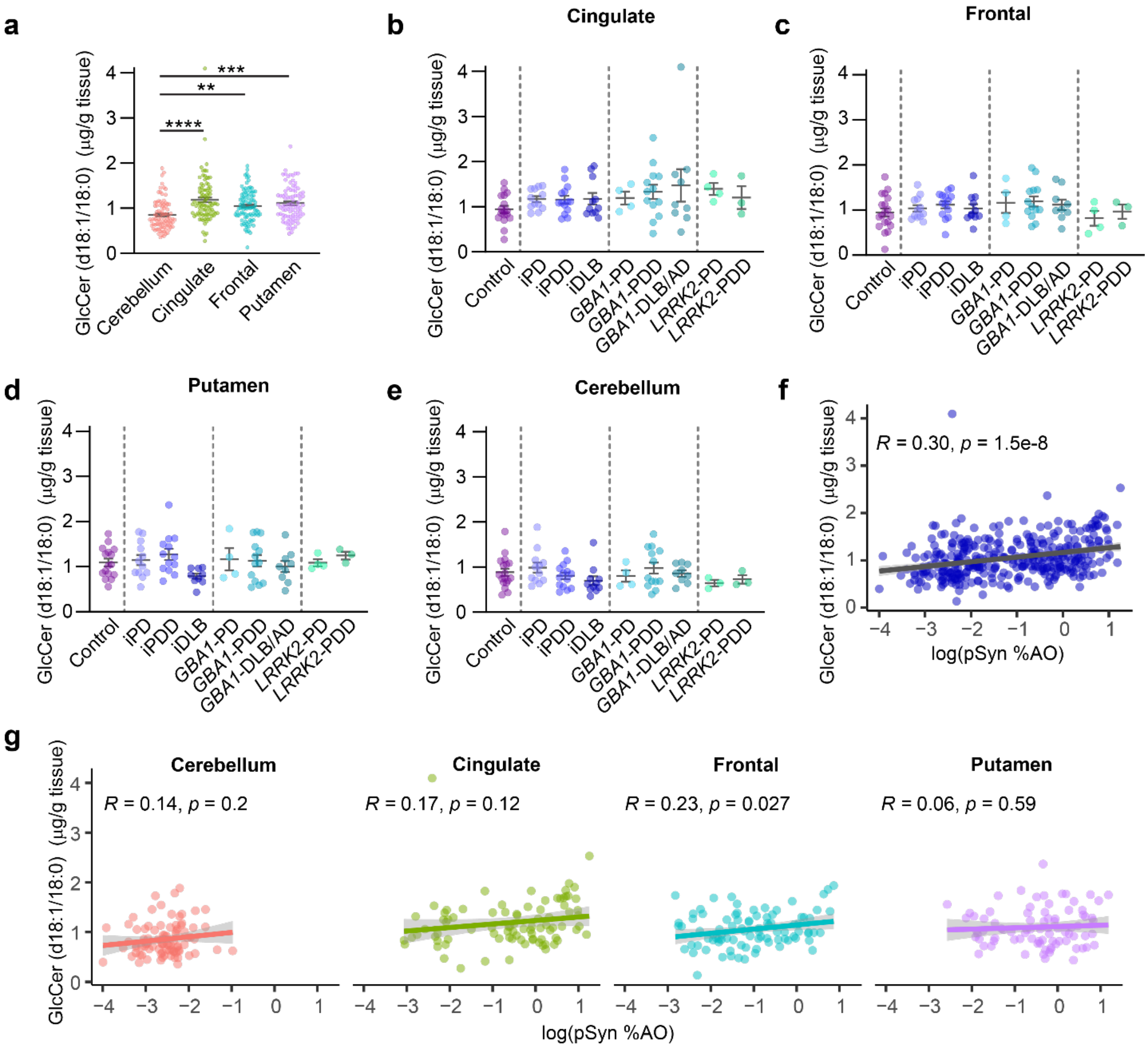

Supplementary Figure 5. GlcCer (d18:1/18:0) isoform in genetic and idiopathic PD (**a**) GlcCer (d18:1/18:0) measures for all cases, separated by brain region. GlcCer species levels are subsequently broken down by neuropathological disease and genetics for each of the four regions: (**b**) cingulate, (**c**) frontal, (**d**) putamen, and (**e**) cerebellum. Bars represent mean +/- S.E.M. with individual values plotted. (**f**) Log normalized pSyn pathology plotted against normalized GlcCer (d18:1/18:0) activity for all samples. (**g**) Log normalized pSyn pathology plotted against GlcCer (d18:1/18:0) levels but broken down by brain region. Lines represent linear regression line of best-fit and shaded area is the 95% confidence interval. Panels **a**, **b**, **c**, **d**, **e**: One-way ANOVA; Tukey's multiple comparison test. *p<0.05, **p<0.01, ***p<0.001, ****p<0.0001.

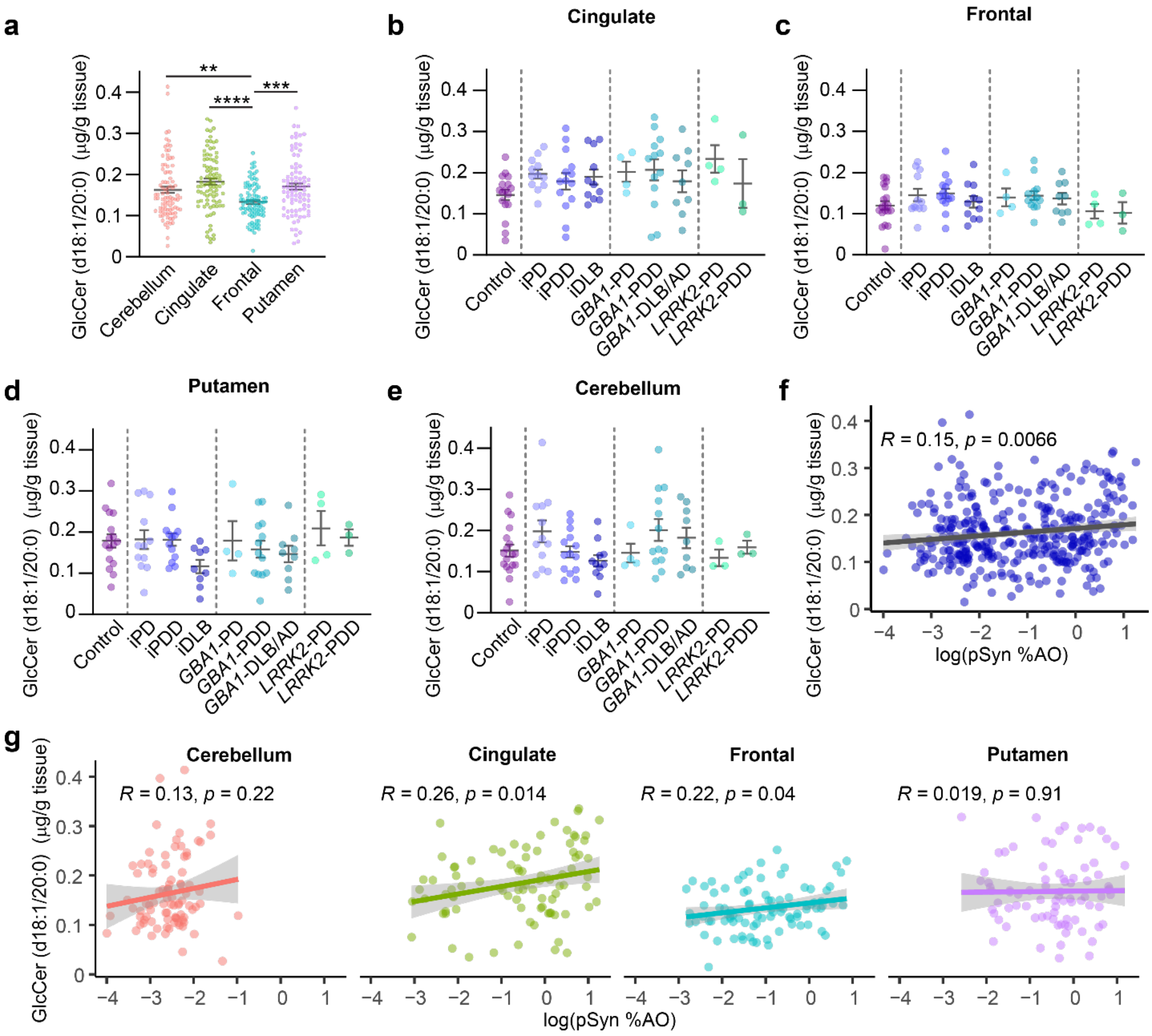

Supplementary Figure 6. GlcCer (d18:1/20:0) isoform in genetic and idiopathic PD (**a**) GlcCer (d18:1/20:0) measures for all cases, separated by brain region. GlcCer species levels are subsequently broken down by neuropathological disease and genetics for each of the four regions: (**b**) cingulate, (**c**) frontal, (**d**) putamen, and (**e**) cerebellum. Bars represent mean +/- S.E.M. with individual values plotted. (**f**) Log normalized pSyn pathology plotted against normalized GlcCer (d18:1/20:0) activity for all samples. (**g**) Log normalized pSyn pathology plotted against GlcCer (d18:1/20:0) levels but broken down by brain region. Lines represent linear regression line of best-fit and shaded area is the 95% confidence interval. Panel **a**: Welch's ANOVA test; Dunnett's T3 multiple comparisons test. **b**, **c**, **d**, **e**: One-way ANOVA; Tukey's multiple comparison test. *p<0.05, **p<0.01, ***p<0.001, ****p<0.0001.

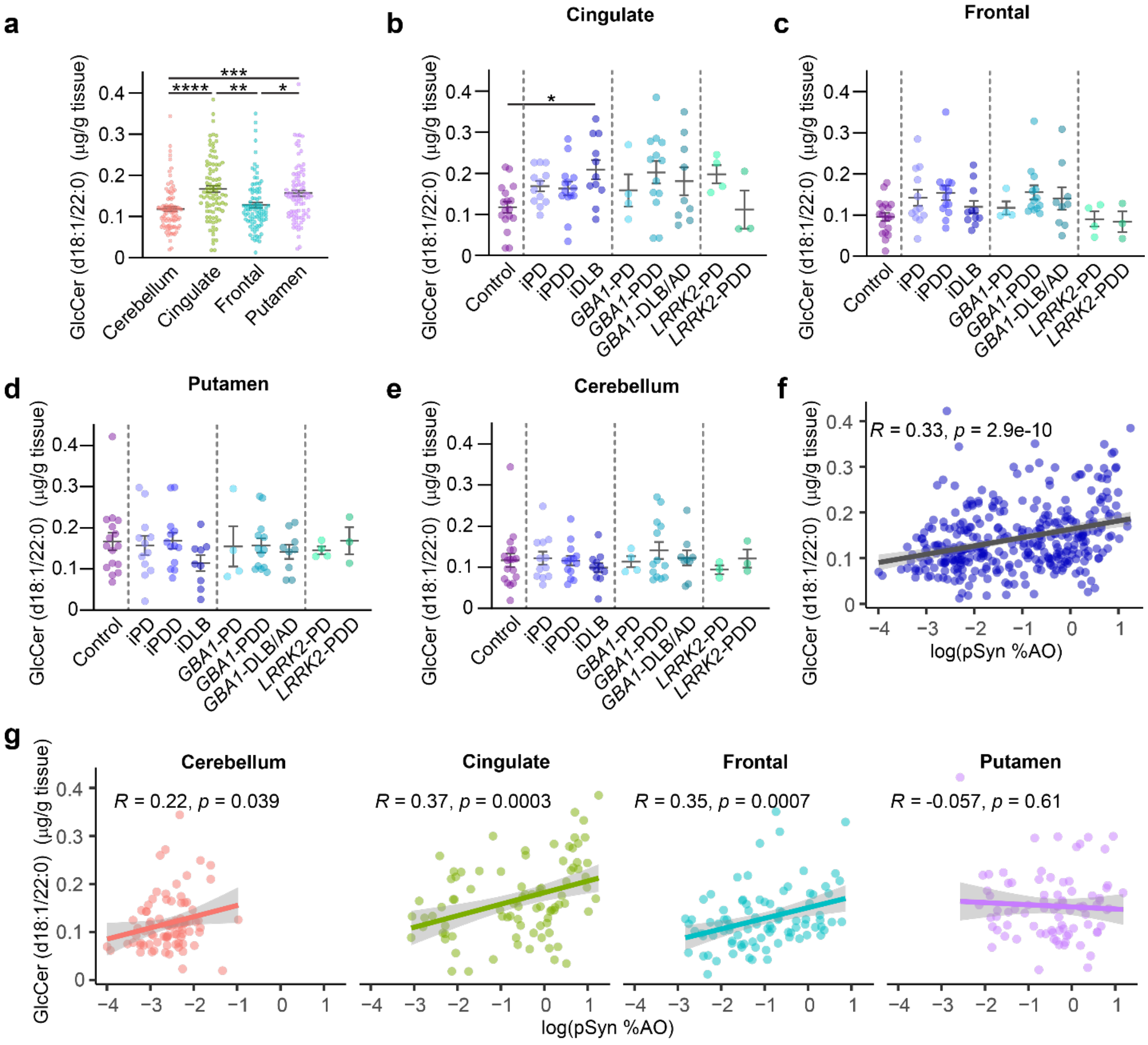

Supplementary Figure 7. GlcCer (d18:1/22:0) isoform in genetic and idiopathic PD (**a**) GlcCer (d18:1/22:0) measures for all cases, separated by brain region. GlcCer species levels are subsequently broken down by neuropathological disease and genetics for each of the four regions: (**b**) cingulate, (**c**) frontal, (**d**) putamen, and (**e**) cerebellum. Bars represent mean +/- S.E.M. with individual values plotted. (**f**) Log normalized pSyn pathology plotted against normalized GlcCer (d18:1/22:0) activity for all samples. (**g**) Log normalized pSyn pathology plotted against GlcCer (d18:1/22:0) levels but broken down by brain region. Lines represent linear regression line of best-fit and shaded area is the 95% confidence interval. Panel **a**: Welch's ANOVA test; Dunnett's T3 multiple comparisons test. **b**, **c**, **d**, **e**: One-way ANOVA; Tukey's multiple comparison test. *p<0.05, **p<0.01, ***p<0.001, ****p<0.0001.

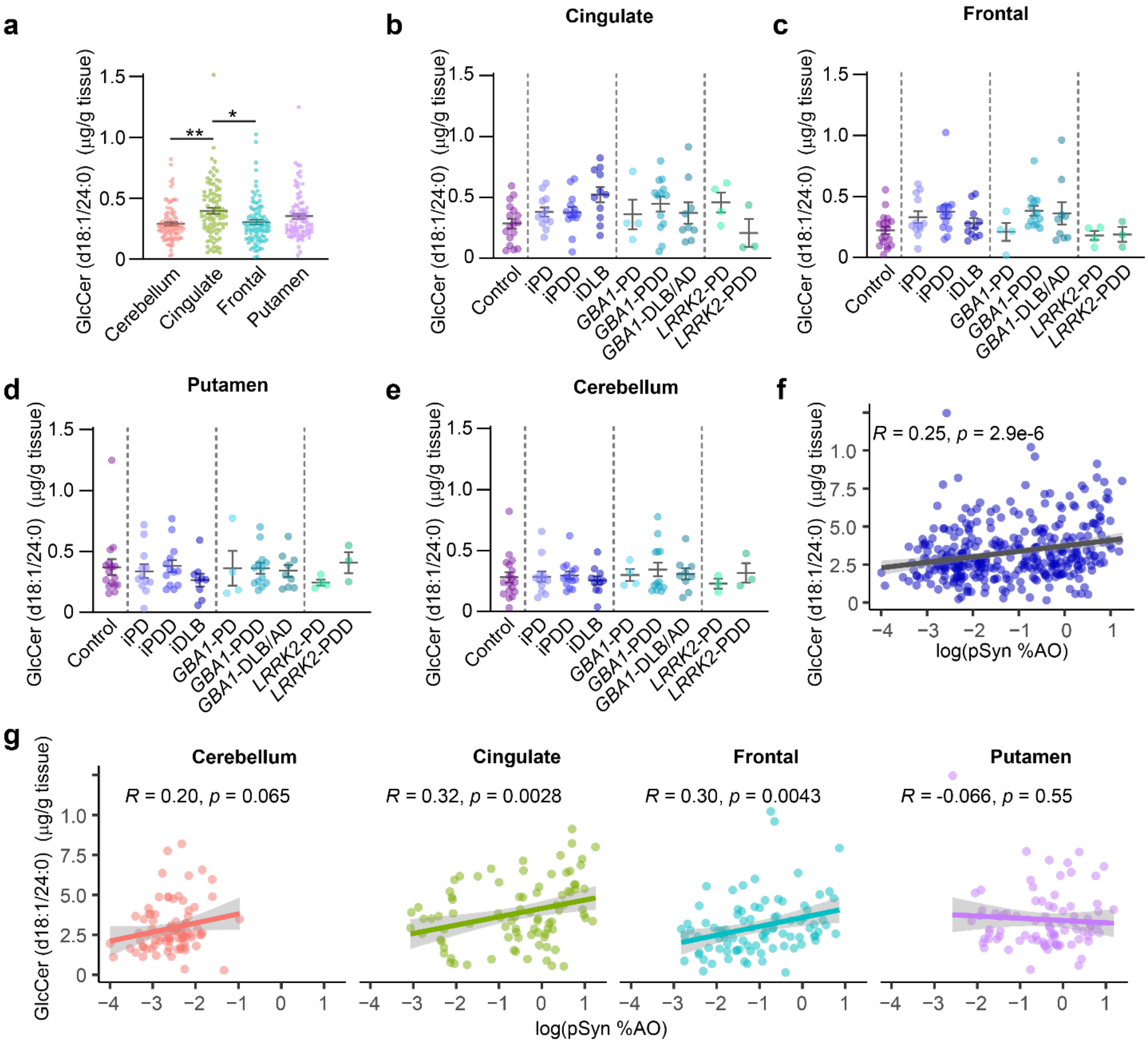

Supplementary Figure 8. GlcCer (d18:1/24:0) isoform in genetic and idiopathic PD (**a**) GlcCer (d18:1/24:0) measures for all cases, separated by brain region. GlcCer species levels are subsequently broken down by neuropathological disease and genetics for each of the four regions: (**b**) cingulate, (**c**) frontal, (**d**) putamen, and (**e**) cerebellum. Bars represent mean +/- S.E.M. with individual values plotted. (**f**) Log normalized pSyn pathology plotted against normalized GlcCer (d18:1/24:0) activity for all samples. (**g**) Log normalized pSyn pathology plotted against GlcCer (d18:1/24:0) levels but broken down by brain region. Lines represent linear regression line of best-fit and shaded area is the 95% confidence interval. Panel **a**: Welch's ANOVA test; Dunnett's T3 multiple comparisons test. **b**, **c**, **d**, **e**: One-way ANOVA; Tukey's multiple comparison test. *p<0.05, **p<0.01, ***p<0.001, ****p<0.0001.

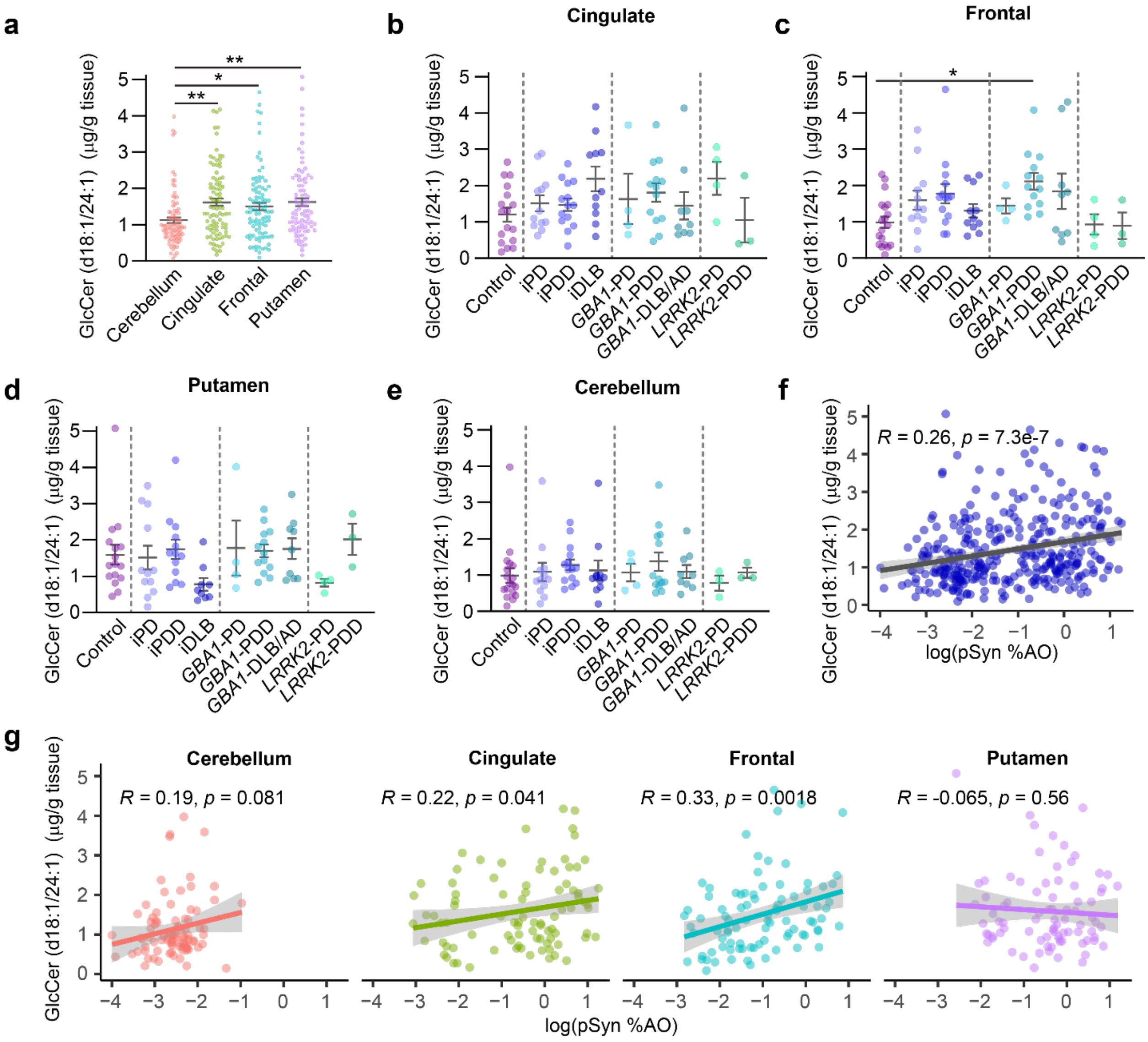

Supplementary Figure 9. GlcCer (d18:1/24:1) isoform in genetic and idiopathic PD (**a**) GlcCer (d18:1/24:1) measures for all cases, separated by brain region. GlcCer species levels are subsequently broken down by neuropathological disease and genetics for each of the four regions: (**b**) cingulate, (**c**) frontal, (**d**) putamen, and (**e**) cerebellum. Bars represent mean +/- S.E.M. with individual values plotted. (**f**) Log normalized pSyn pathology plotted against normalized GlcCer (d18:1/24:1) activity for all samples. (**g**) Log normalized pSyn pathology plotted against GlcCer (d18:1/24:1) levels but broken down by brain region. Lines represent linear regression line of best-fit and shaded area is the 95% confidence interval. Panel **a**: Welch's ANOVA test; Dunnett's T3 multiple comparisons test. **b**, **c**, **d**, **e**: One-way ANOVA; Tukey's multiple comparison test. *p<0.05, **p<0.01, ***p<0.001, ****p<0.0001.

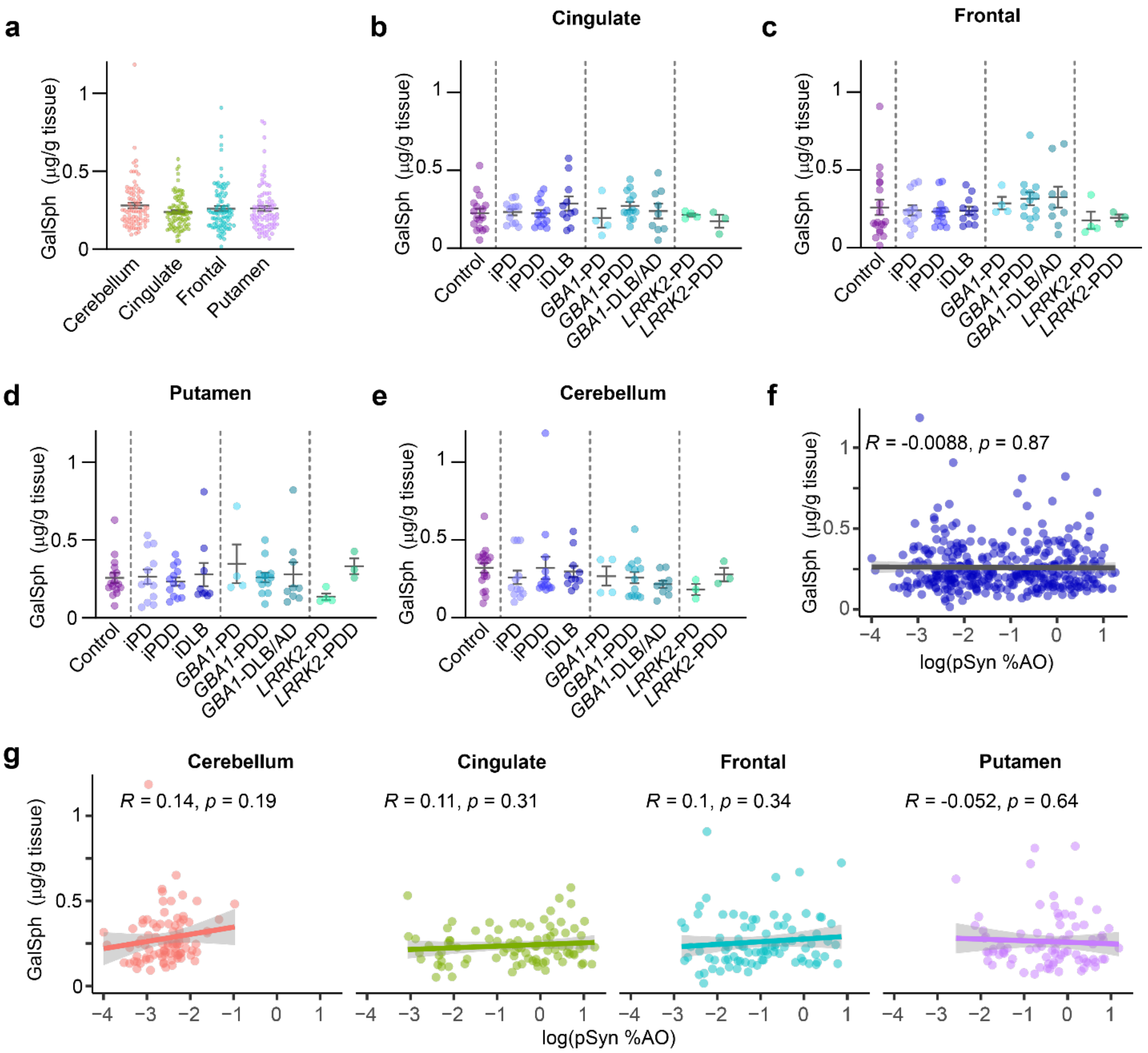

Supplementary Figure 10. GalSph (d18:1) in genetic and idiopathic PD (**a**) GalSph measures for all cases, separated by region. GalSph levels are subsequently broken down by neuropathological disease and genetics for each of the four regions: (**b**) cingulate, (**c**) frontal, (**d**) putamen, and (**e**) cerebellum. Bars represent mean +/- S.E.M. with individual values plotted. (**f**) Log normalized pSyn pathology plotted against GalSph levels for all samples. (**g**) Log normalized pSyn pathology plotted against GalSph levels but broken down by brain region. Lines represent linear regression line of best-fit and shaded area is the 95% confidence interval. Panels **a**, **b**, **c**, **d**, **e**: One-way ANOVA; Tukey's multiple comparison test.

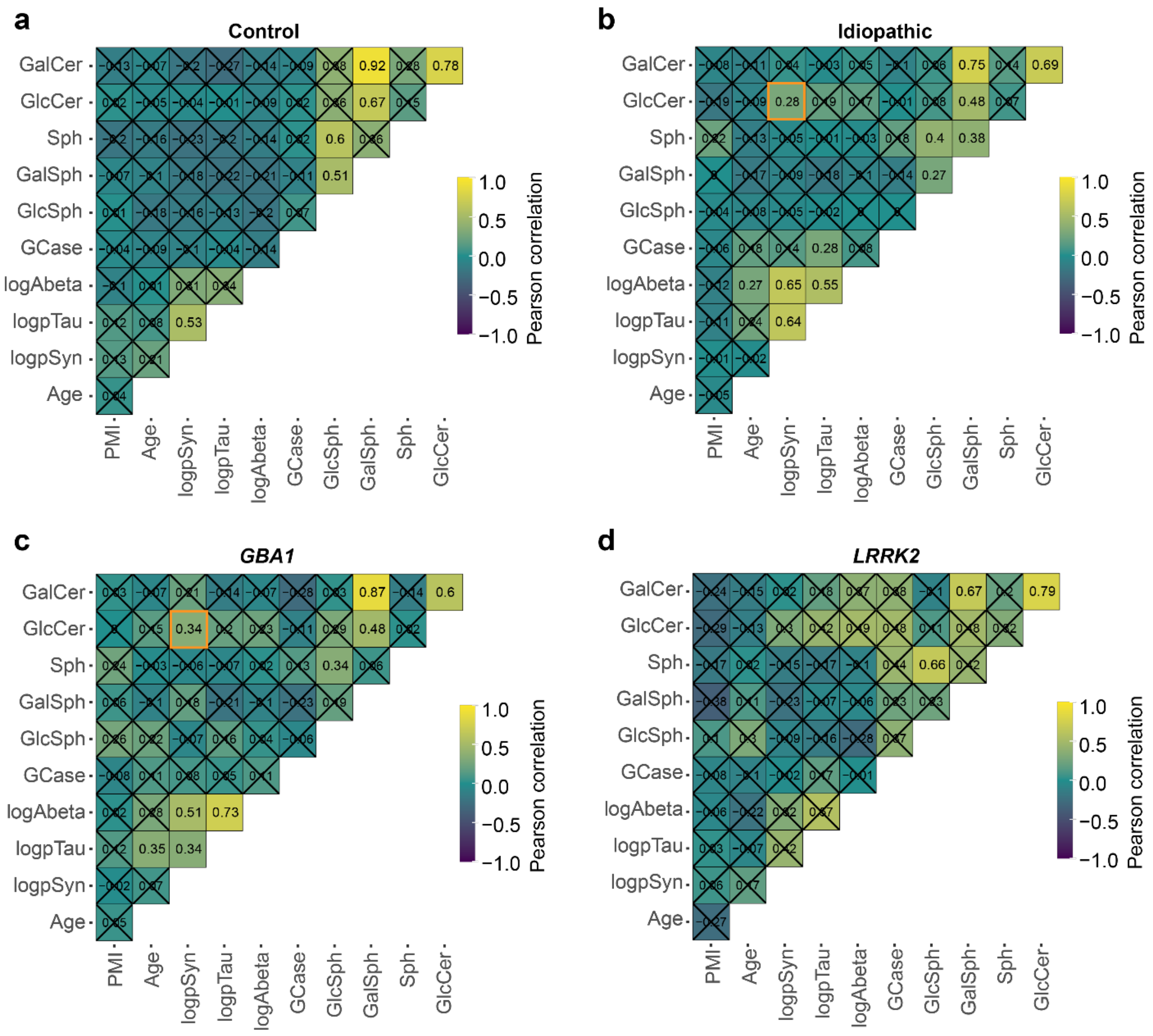

Supplementary Figure 11. Overall relationships between neuropathology, GCase, and lipids by disease Pearson's correlations between each of the different measure factors, including age, post-mortem interval (PMI), pathology, GCase activity and lipids are plotted here separately for control (**a**), idiopathic (**b**), *GBA1* (**c**), and *LRRK2*-linked PD (**d**). Several of the protein pathologies correlate with each other; several lipid levels also correlate with each other. There is minimal correlation with pathologies and lipids within each disease group, with the exception of the correlation of GlcCer with logpSyn in idiopathic and *GBA1*-PD tissue.

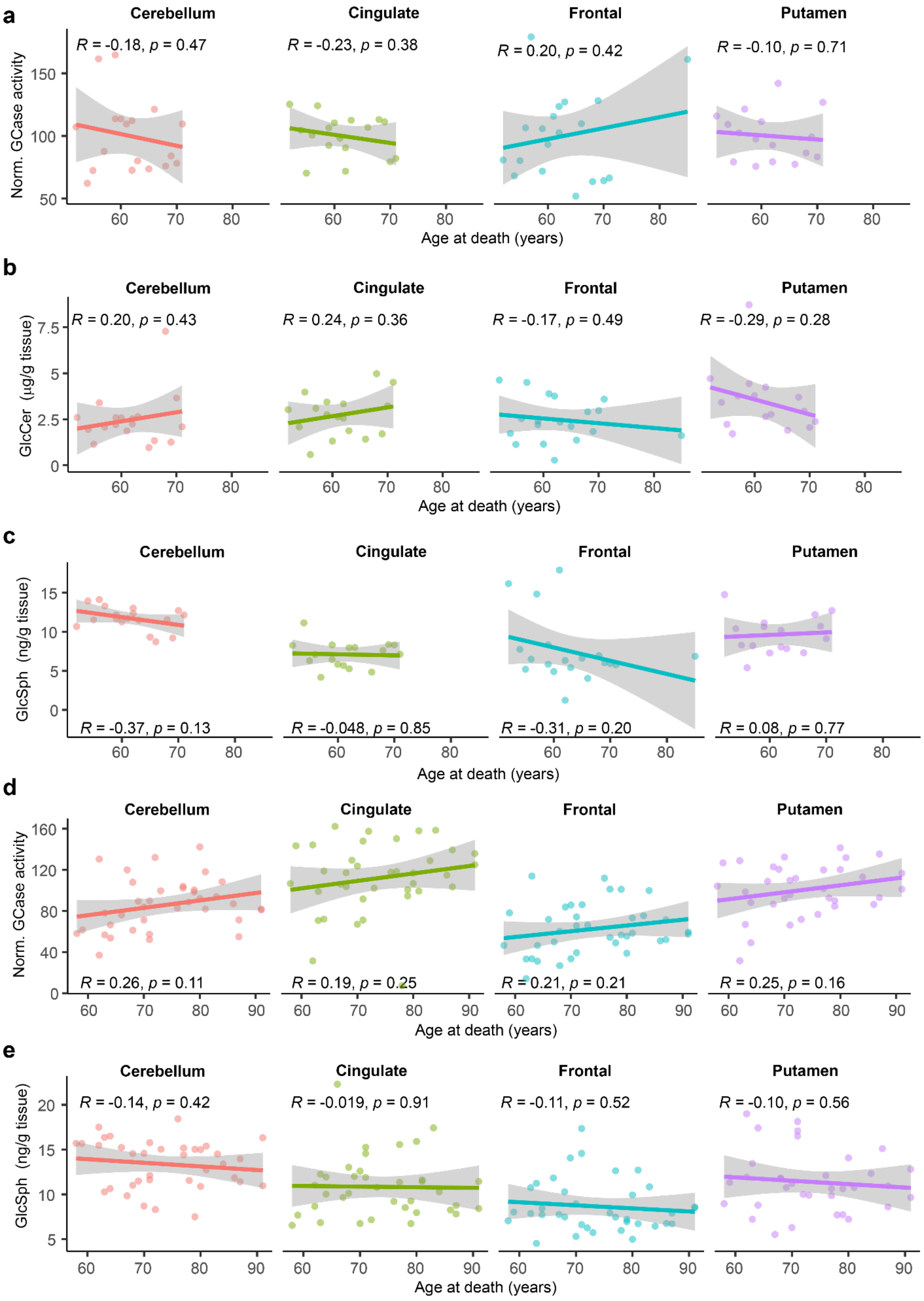

a
Cerebellum
Cingulate
Frontal
Putamen
R = -0.18, p = 0.47
R = -0.23, p = 0.38
R = 0.20, p = 0.42
R = -0.10, p = 0.71
Norm. GCase activity
Age at death (years)
b
Cerebellum
Cingulate
Frontal
Putamen
R = 0.20, p = 0.43
R = 0.24, p = 0.36
R = -0.17, p = 0.49
R = -0.29, p = 0.28
GlcCer (µg/g tissue)
Age at death (years)
c
Cerebellum
Cingulate
Frontal
Putamen
R = -0.37, p = 0.13
R = -0.048, p = 0.85
R = -0.31, p = 0.20
R = 0.08, p = 0.77
GlcSph (ng/g tissue)
Age at death (years)
d
Cerebellum
Cingulate
Frontal
Putamen
R = 0.26, p = 0.11
R = 0.19, p = 0.25
R = 0.21, p = 0.21
R = 0.25, p = 0.16
Norm. GCase activity
Age at death (years)
e
Cerebellum
Cingulate
Frontal
Putamen
R = -0.14, p = 0.42
R = -0.019, p = 0.91
R = -0.11, p = 0.52
R = -0.10, p = 0.56
GlcSph (ng/g tissue)
Age at death (years)

Supplementary Figure 12. Correlations with age Age at death for all control subjects for each of the for regions collected was plotted against normalized GCase activity (**a**), total GlcCer levels (**b**), or GlcSph levels (**c**). No significant correlations were observed. Age at death was also examine for idiopathic PD/PDD/DLB plotted against normalized GCase activity (**d**) and GlcSph levels (**e**). No significant correlations were observed. Lines represent linear regression line of best-fit and shaded area is the 95% confidence interval.

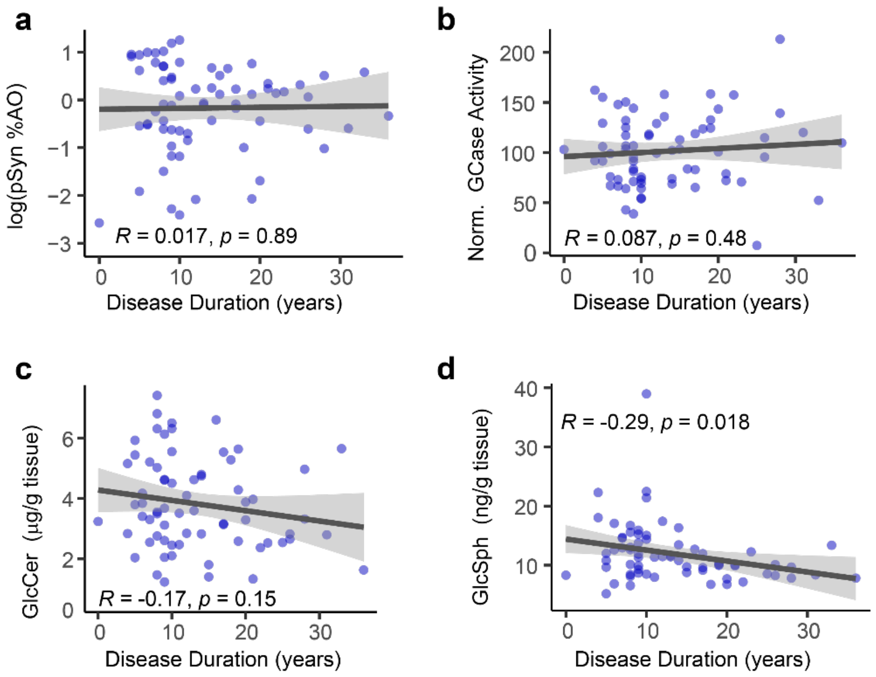

Supplementary Figure 13. Correlations with disease duration and cingulate cortex measures Disease duration for all PD/PDD/DLB subjects was plotted against log normalized pSyn pathology (**a**), normalized GCase activity (**b**), total GlcCer levels (**c**), or GlcSph levels (**d**). The only significant relationship observed was a slight negative correlation between GlcSph levels and disease duration (Panel D). Lines represent linear regression line of best-fit and shaded area is the 95% confidence interval.

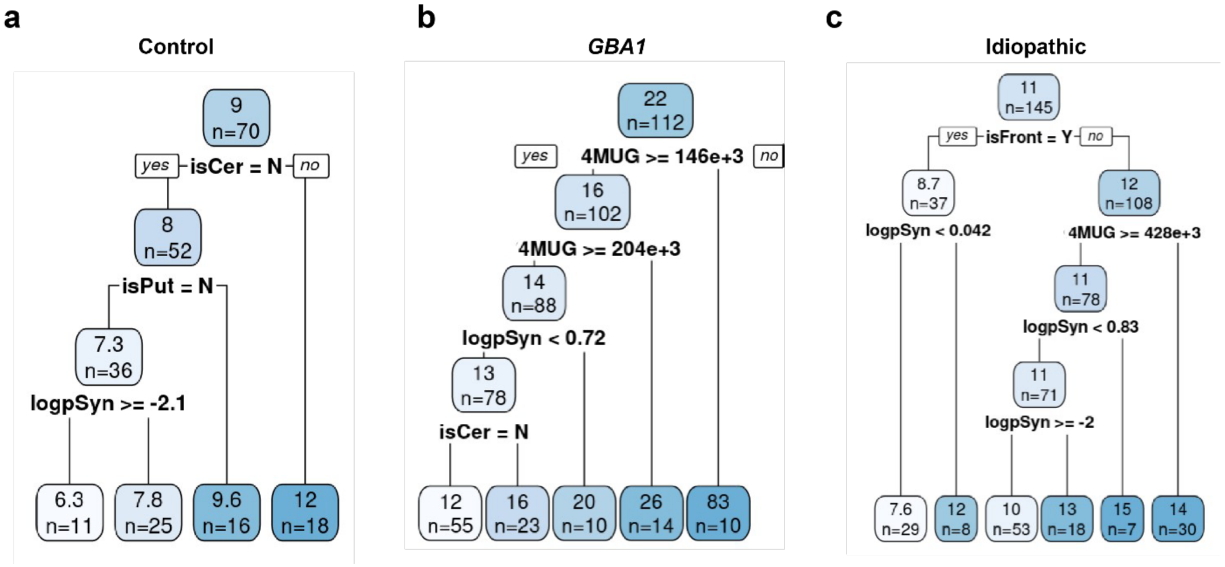

Supplemental Figure 14. Decision tree analysis of variables influencing GlcSph levels Three decision trees were generated with (**a**) control, (**b**) *GBA1* and (**c**) idiopathic groups. The root node indicates the average GlcSph value across all samples (sample size indicated below) and each node is then split into left and right sub-nodes. Optimal split variable and associated cut point were shown in each tree up to maximum depth of 5. Tracing the trajectories from root to leaf exhibits insights of the joint interaction pattern of different splitting variables in driving GlcSph. For example, the right path of the decision tree in (**c**) idiopathic group, 4-MUG and pSyn were interacting with one another in non-frontal regions to regulate GlcSph.
